# Oxidative stress responses in *Ehrlichia*-induced sepsis: mechanisms and implications in pathogenesis

**DOI:** 10.1128/mbio.00499-25

**Published:** 2025-06-09

**Authors:** Aditya Kumar Sharma, Nahed Ismail

**Affiliations:** 1 Department of Pathology, College of Medicine, University of Illinois at Chicago 12247, Chicago, Illinois, USA; 2 Department of Life Sciences, School of Biosciences and Technology, Galgotias University 357911, Greater Noida, Uttar Pradesh, India; The Ohio State University, Columbus, Ohio, USA

**Keywords:** oxidative stress, *Ehrlichia*, mitochondrial damage, NRF2 pathway, anti-oxidative response, sepsis, inflammation, liver injury

## Abstract

The host immune system uses reactive oxygen species (ROS) and reactive nitrogen species to build up an oxidative response as a host defense mechanism against infectious agents. However, uncontrolled ROS causes excessive inflammation and oxidative stress, which is counteracted by host antioxidant responses. Oxidative stress is recognized as an imbalance between the production of oxidants such as ROS and antioxidant defenses, which account for organ damage following severe infection with bacterial pathogens. *Ehrlichia* is an obligatory intracellular Gram-negative bacterial pathogen that causes human monocytic ehrlichiosis (HME), a potentially life-threatening tick-borne emerging infectious disease. Patients with severe HME develop sepsis that progresses to multi-organ failure. *Ehrlichia* develops several immune evasion strategies that cause oxidative stress and tissue damage. This review discusses various mechanisms that *Ehrlichia* employs to counteract host anti-oxidative responses, including the complex interplay among oxidative stress, mitochondrial damage, and endoplasmic stress during *Ehrlichia*-induced sepsis. Understanding these immune evasion strategies is important for the rational development of targeted therapy, not only for severe HME but also for other infectious and non-infectious diseases where oxidative stress is a key mechanism in pathogenesis.

## HUMAN MONOCYTIC EHRLICHIOSIS

Human monocytic ehrlichiosis (HME) is an emerging tick-borne zoonotic infectious disease caused by *Ehrlichia* species. Patients with HME present with an initial non-specific clinical manifestation, which is often undiagnosed or misdiagnosed, partially due to a lack of highly sensitive and specific diagnostic tests, resulting in delayed treatment. Initial treatment with doxycycline can be effective; however, late treatment is frequently ineffective in preventing disease progression and development of complications such as sepsis and multi-organ failure (1). The liver is the main initial site of infection and pathology in ehrlichiosis. Acute liver injury is the main cause of death in HME as it usually leads to sepsis and multi-organ failure, if left untreated. Notably, extensive liver damage and fatal infection in immunocompetent patients with HME is associated with a low bacterial burden in blood as well as hyperactivation of macrophages, a condition known as hemophagocytic lymphohistiocytic syndrome, suggesting that disease severity is due to immunopathology. *E. chaffeensis*, the main causative agent of HME, is an obligate intracellular Gram-negative bacterium that lacks lipopolysaccharide and peptidoglycan. Other *Ehrlichia* species that also cause HME include *E. ewingii*, *E. canis*, and *E. muris* subsp. *Eauclairensis*. These *Ehrlichia* species differ in their virulence, and that may account for the various infection outcomes in patients with HME as well as in animal models of ehrlichiosis. *Ehrlichia* sp. HF565, previously referred to as *Ixodes ovatus Ehrlichia* (IOE) and currently named *E. japonica,* has been isolated from *Ixodes ovatus* ticks in Japan and causes acute to fatal infections in a murine model of fatal ehrlichiosis (2).

Toward understanding the pathogenesis of severe and potentially fatal ehrlichiosis, we have employed murine models of mild and fatal ehrlichiosis caused by infection of immunocompetent C57BL/6 mice with mildly virulent *E. muris* and highly virulent *E. japonica* (previously called *Ixodes Ovatus Ehrlichia*), respectively. These models recapitulate the clinical, pathological, and laboratory findings in patients with mild and fatal ehrlichiosis. Virulent *E. japonica* infection causes acute and lethal liver injury characterized by hepatic necrosis, microvesicular steatosis, and hepatocellular steatosis, which is associated with excessive hepatic inflammation as well as systemic cytokine and chemokine storm. This pathology is associated with ineffective bacterial clearance, resulting in 100% mortality of infected mice at 8–12 days post-infection (p.i.). By contrast, infection of mice with *E. muris* results in mild and self-limited diseases characterized by hepatosplenomegaly, minimal liver pathology, and effective clearance of bacteria at day 9 p.i (3). In the following sections, we will provide an overview of oxidative stress and anti-oxidative response in ehrlichiosis using these model systems.

## OXIDATIVE STRESS IN EHRLICHIOSIS

ROS encompasses a group of highly reactive and unstable molecules containing oxygen that are metabolic byproducts of different cellular processes (4). Major ROS include superoxide anion (O_2_**^−^**), hydroxyl radical (OH), hydrogen peroxide (H_2_O_2_), singlet oxygen (O_2_), peroxyl radical (ROO**·**), hypochlorous acid (HOCl), and alkoxyl radical (RO**·**). These molecules are formed by reduction-oxidation (redox) reactions or by electronic excitation. Oxidative stress is the result of an imbalance between the production of oxidants such as ROS and antioxidant defenses and has been shown to play a role in the pathogenesis of several diseases, such as atherosclerosis, Alzheimer’s disease, chronic obstructive pulmonary disease, cancer, and certain infectious diseases (4).

The extent to which oxidative stress contributes to the pathology of these diseases is variable and depends on the complexity and nature of the disease process. Infections with facultative intracellular bacteria such as *Salmonella* trigger upregulation of two periplasmic proteins—SodCI and SodCII—which contribute to the virulence of *Salmonella* (5). Similarly, in *Helicobacter pylori,* the *sodB* gene is important for virulence and the organism’s ability to survive inside the mouse stomach, which is an oxidative-rich environment (6). Unlike facultative intracellular bacteria, obligatory intracellular bacteria such as *Ehrlichia* lack many genes that mediate oxidative stress resistance. Thus, *Ehrlichia* relies on the host cell’s antioxidant defenses to protect itself from oxidative damage (3). *E. chaffeensis* encodes two functional enzymes, GshA and GshB, to synthesize glutathione, which confers *Ehrlichia* resistance to oxidative stress. The expression of *gshA* and *gshB* is upregulated by *CtrA*, a global transcriptional regulator that binds to promoters of *gshA* and *gshB*. CtrA also binds to the promoter region of *bolA* (stress-induced morphogen) and *surE* (stationary phase survival protein) (7). Infection of macrophages with *E. chaffeensis* triggers the upregulation of *gshA* and *gshB* upon oxidative stress as a mechanism that enables intracellular bacterial survival (8). Other studies have shown that *E. chaffeensis* expresses an outer membrane invasion protein EtpE, which facilitates bacterial entry and inhibits ROS-mediated oxidative stress through binding to DNase X, a receptor expressed on bone marrow-derived macrophages (9). Notably, *E. chaffeensis* does not prevent the production of ROS in neutrophils, as DNase X is not expressed in these cells (10).

Iron plays a crucial role in ROS generation as it can catalyze ROS formation through the Fenton reaction. Studies have shown that the lack of access to cytoplasmic iron compromises the survival of *E. chaffeensis* within infected human monocytes, suggesting that *E. chaffeensis* exploits iron metabolism for its intracellular survival (11). Other studies suggested that *Ehrlichia* tandem repeat proteins 32 (TRP32), a type I secretion system effector, may recruit host ferritin light polypeptide to stabilize the labile iron pool, thus enabling *Ehrlichia* replication (12). Other studies have shown that dogs infected with *E. canis* develop a state of redox imbalance by increasing the concentration of nitrite/nitrate, lipid peroxidation, oxidation protein products, and glutathione reductase activity (13). Indeed, dogs naturally infected with *E. canis* showed changes in iron metabolism and had an altered oxidant environment (14).

## MITOCHONDRIAL DAMAGE AS A POTENTIAL MECHANISM OF INFLAMMASOME ACTIVATION AND OXIDATIVE STRESS IN EHRLICHIOSIS

Mitochondrial dysfunction and oxidative stress are interlinked. Mitochondrial dysfunction and associated oxidative stress have been linked to several pathological conditions such as Parkinson’s, Alzheimer’s disease, metabolic disorders, and aging. Mechanistically, oxidative stress was found to be responsible for the change in β-amyloid to aggregated β-amyloid, one of the key pathogenic mechanisms in Alzheimer’s disease (15). Furthermore, oxidative stress leads to increased phosphorylation of tau protein, leading to microtubule instability and self-assembly as well as its aggregation, another key mechanism that accounts for the development of Alzheimer’s disease (15, 16).

Recent studies by our group have shown that *E. japonica, a* highly virulent *Ehrlichia* species that causes severe and fatal ehrlichiosis in immunocompetent mice mimicking severe HME, infects and replicates in hepatocytes. Hepatocytes infected with *E. japonica* exhibit increased levels of ROS, altered mitochondrial membrane potential, and abnormal mitochondrial morphology (swollen matrices and damaged cristae), suggesting that virulent *Ehrlichia* triggers mitochondrial dysfunction. These events likely contribute to reduced cell viability and exacerbated cell death in the form of apoptosis and necrosis following severe *Ehrlichia* infection in mice and humans (3). Elevated ROS and mitochondrial damage in *E. japonica-*infected hepatocytes correlate with an increased generation of oxidized cardiolipin, another marker for mitochondrial dysfunction (3). Cardiolipin is a major membrane phospholipid primarily found in the inner mitochondrial membrane, where it undergoes oxidation with selective modification in its fatty acyl chains during mitochondrial oxidative stress. Oxidized cardiolipin facilitates the release of cytochrome c and activation of pro-apoptotic proteins, triggering the initiation of the apoptotic cascade (17). Mitochondrial damage has also been detected in *E. japonica-*infected primary bone marrow-derived macrophages, although it is unclear whether this process also resulted in the expression of oxidized cardiolipin (18). Notably, the expression of oxidized cardiolipin in *E. japonica*-infected hepatocytes has been linked to the activation of caspase 11, which causes inflammatory cell death, known as pyroptosis, and liver damage. Although the exact mechanism that accounts for mitochondrial damage in hepatocytes and macrophages following infection with virulent *Ehrlichia* remains unclear, our studies suggest that blocked autophagy flux and mitophagy (elimination of damaged mitochondria through autophagy) may be responsible, in part, for the observed mitochondrial damage in infected cells. In macrophages and hepatocytes, *E. japonica* blocks autophagy flux via Myeloid differentiation primary response 88 (MYD88) interaction with the mechanistic target of rapamycin complex 1 (mTORC1). MYD88 acts as an adapter molecule, connecting signals from outside or inside the cells, particularly for Toll-like receptors and interleukin-1 (IL-1) receptors, and thus mediates activation of innate immune responses. mTORC1 is a key regulator of cell growth and metabolism by sensing nutrients, energy, and growth factors. Activation of mTORC1 mediates cellular anabolism as it promotes the formation of macromolecules like proteins, lipids, and nucleic acids while suppressing catabolism. mTORC1 is also a negative regulator of autophagy. Genetic deficiency of MYD88 signaling or pharmacologic inhibition of mTORC1 activation restores autophagy flux and enhances mitophagy in macrophages. Interestingly, the accumulation of dysfunctional mitochondria within host cells can also inhibit autophagy in a positive feedback loop (18, 19). Alteration of mitochondrial metabolism was also suggested as another potential mechanism that causes mitochondrial damage following infection of macrophages with *E. chaffeensis,* the main human pathogen causing HME. Infection of macrophages with *E. chaffeensis* triggered elevated expression of mitochondrial-associated genes such as san (N-(alpha) acetyltransferase 50), *Cht11* (chitinase 11), *Uck2* (uridine/cytidine kinase), *Echs1* (enoyl-CoA hydratase), *whd* (carnitine palmitoyl transferase 1), *Ccdc58* (coiled-coil domain-containing protein 58), and *Apop1* (apoptogenic 1) in infected macrophages (20). Proteins encoded by these genes are involved in mitochondrial homeostasis. For example, SAN is responsible for producing phosphatidic acid, whereas UCK2 generates CTP (cytidine triphosphate) from CMP (cytidine monophosphate), which are key substrates required for the biosynthesis of cardiolipin. ECHS1 and WHD are involved in the formation of acetyl-CoA, a metabolic product that facilitates mitochondrial oxidative phosphorylation. CCDC58 is associated with MRPS28 (mitochondrial ribosomal protein S28) and SSBP1 (single-stranded DNA-binding protein). APOP1 has a role in apoptosis that triggers cytochrome C release through the permeability transition pore (20).

Two mitochondrial proteins, PINK and PARKIN, are crucial for maintaining the cellular mitochondrial quality. PARKIN is a RING-between-RING type E3 ubiquitin ligase, while PINK1 is a mitochondrial-targeted serine-threonine kinase (21). We have recently shown that the infection of murine primary hepatocytes with virulent *E. japonica* resulted in reduced expression of PINK1 and PARKIN at protein and mRNA levels. In addition, *E. japonica* inhibits mitochondrial autophagy (i.e., mitophagy) in hepatocytes as well as primary bone marrow-derived macrophages. *Ehrlichia*-induced downregulation of PINK1 and PARKIN and blocked mitophagy suggest a defective elimination of damaged mitochondria in infected hepatocytes (3). Further studies indicated that the accumulation of damaged mitochondria in infected cells, such as macrophages, is linked to excessive activation of canonical (marked by caspase 1 activation) and non-canonical (marked by caspase 11 activation) inflammasome pathways. Activation of both canonical and non-canonical inflammasome pathways and subsequent secretion of inflammasome-dependent cytokines such as IL-1β and IL-18 is responsible for immunopathology, dysregulated innate and adaptive immune responses, and fatal outcome following infection of mice with *E. japonica*. Indeed, IL-18R-deficient mice infected with *E. japonica* exhibited attenuated liver damage, decreased bacterial burden in different organs, and developed protective immunity, which resulted in mild disease and mice survival compared to severe disease and fatal outcome in *E. japonica-*infected wild-type mice.

Further *in vitro* studies showed that the activation of caspase 11 in *E. japonica-*infected hepatocytes is positively regulated by both MYD88 and type I IFN (IFN-I) receptor (IFNAR) signaling. MYD88-mediated caspase 11 activation in hepatocytes triggers cleavage of Gasdermin D, leading to pyroptosis and inflammatory cell death (18). Although the exact ligand activating canonical and non-canonical inflammasome pathways during severe ehrlichiosis is not known, our studies suggest that mitochondria-derived damage-associated molecular patterns (DAMPs) produced in *E. japonica*-infected macrophages and hepatocytes are a major molecule triggering inflammasome activation.

Although our studies showed a strong link between activation of caspase 1 and caspase 11 following severe *E. japonica* infection and the development of extensive liver damage and fatal outcome, the following evidence suggests that caspase 11, but not caspase 1, is the key factor that accounts for *Ehrlichia*-induced acute liver injury and fatal infection. First, we detect the expression of caspase 1 and caspase 11 in the liver of lethally (*E. japonica*) and non-lethally (*E. muris*) infected mice during the course of infection. However, we only detect expression of caspase 11 in the lethally infected mice at late stages of infection (days 5 and 7 post-infection) (22). The lack of activation of caspase 11 following mild *E. muris* infection correlates with protection and survival. Second, resistance of both MYD88^−/−^- or IFNAR^−/−^-deficient mice to fatal ehrlichiosis and survival of these mice following *E. japonica* infection was associated with the absence of caspase 11 activation. Third, activation of caspase 11 in primary hepatocytes infected *in vitro* with *E. japonica* resulted in hepatocyte pyroptosis, that is, inflammatory cell death, which further exacerbated following the addition of recombinant IFNβ and IFNAR signaling (23). These data suggest a deleterious or pathogenic role of hepatic caspase 11 during *Ehrlichia* infection.

By contrast, several *in vivo* and *in vitro* studies suggested that active caspase 1 is hepatoprotective. For example, mice lacking caspase 1 and infected with virulent *E. japonica* developed extensive liver damage, had a higher bacterial burden in the liver, and succumbed to fatal infection at earlier time points after infection when compared to similarly infected wild-type mice (24). In addition, *in vitro* studies showed that MYD88 signaling in hepatocytes infected with *E. japonica* attenuated the expression of caspase 1. MYD88-mediated downregulation of caspase 1 activation in infected hepatocytes correlated with cell survival (24). Furthermore, the lack of MYD88 in *E. japonica*-infected hepatocytes upregulated the expression of active caspase 1, which caused activation of caspase 3 and triggered apoptotic cell death. These data suggest that activation of caspase 1 in hepatocytes is hepato-protective as it promotes cell survival via inhibition of caspase 3, a key mechanism of apoptotic cell death. These *in vitro* data explain the heightened susceptibility of caspase 1-deficient mice to severe and fatal ehrlichiosis compared to wild-type mice.

## DYSFUNCTIONAL NRF2 SIGNALING AS A POTENTIAL MECHANISM THAT ACCOUNTS FOR THE DEVELOPMENT OF SEVERE EHRLICHIOSIS

NRF2 (nuclear factor erythroid 2-related factor 2) signaling is a critical defense mechanism that protects cells from oxidative stress and inflammation. NRF2 is the transcription factor that regulates the expression of detoxifying enzymes, cytoprotective proteins, and various antioxidant proteins (25). NRF2 is a cap’n’collar-basic leucine zipper (CNC-bZIP) transcription factor consisting of critical functional domains: NEH1, NEH2, NEH3, NEH4, NEH5, NEH6, and NEH7. These different domains have distinct functions, such as NEH1, which facilitates DNA binding and forms a heterodimer with MAF proteins, allowing NRF2 to bind to AREs (antioxidant response elements). NEH2 interacts with KEAP1 (Kelch-like ECH-associated protein 1) in the cytoplasm, which regulates NRF2 stability by targeting it for ubiquitination and proteasomal degradation under normal conditions. NEH3, NEH4, and NEH5 domains function as transcriptional activation domains. NEH6 comprises degradation motifs that modulate NRF2 degradation via alternative pathways, while NEH7 suppresses NRF2 activity through interaction with RXRα (retinoic X receptor alpha) (26). NRF2 is a short-lived protein, and its stability inside the cytoplasm depends upon KEAP1 protein, an E3 ubiquitin ligase substrate that targets NRF2 for ubiquitination and proteasomal degradation (25). Under normal conditions, NRF2 is bound to its inhibitor, KEAP1, in the cytoplasm, leading to its ubiquitination and degradation by the proteasome. However, upon oxidative stress, KEAP1 undergoes conformational changes, releasing NRF2, which then translocates into the nucleus. As NRF2 translocates to the nucleus, NRF2 binds to AREs in the promoter regions of the target genes. The NRF2/KEAP1 axis is considered a pivotal point in cellular antioxidant defense and survival pathways, where NRF2 controls the expression of over 1,000 cytoprotective genes. KEAP1, a cytoplasmic repressor of NRF2, undergoes various post-translational modifications. Under oxidative or electrophilic stress, oxidation, alkylation, or S-nitrosylation of cysteine residues impair KEAP1’s ability to target NRF2 for ubiquitination. This disruption results in the stabilization and nuclear translocation of NRF2, leading to cellular antioxidant response (27). In addition, it has been shown that NRF2 is phosphorylated by protein kinase c at serine 40 residue. While this mutation did not affect NRF2 binding to AREs *in vitro*, it caused the dissociation of the NRF2-KEAP1 complex, leading to NRF2 stabilization and nuclear translocation (28). Similar findings showed the importance of casein kinase-mediated NRF2 phosphorylation for nuclear translocation and its transcription function in neuroblastoma (29). Furthermore, the NRF2-KEAP1 complex is also disrupted by acetyltransferase p300, which interacts with and acetylates NRF2, preventing KEAP1-mediated NRF2 ubiquitination and degradation (30). This underscores the crucial role of acetylation in regulating the NRF2-KEAP1 signaling axis.

Nuclear translocation of NRF2 triggers the expression of several antioxidant genes—NAD(P)H quinone oxidoreductase 1 (NQO1), glutathione peroxidase 3 (GPX3), glutathione peroxidase 4 (GPX4), and thioredoxin reductase 1 (TXNRD1), leading to various antioxidant responses that protect the cells against oxidative stress. NQO1 catalyzes the two-electron reduction of quinones to hydroquinones and prevents quinones from undergoing one-electron reduction to form semiquinones that react with oxygen to generate superoxide, thereby NQO1 prevents ROS formation. GPX3 and GPX4 help reduce hydrogen peroxide (H_2_O_2_) and lipid hydroperoxides using glutathione (GSH) as a reducing agent, protecting cells and tissues from oxidative damage caused by H_2_O_2_. TXNRD1 regulates redox homeostasis and effectively neutralizes ROS, including H_2_O_2_ and lipid peroxides (31).

Human studies have shown a key role of NRF2 activation in protecting against infections with Gram-negative bacteria such as non-typeable *Haemophilus influenzae* and *Pseudomonas aeruginosa*. NRF2 signals were associated with enhanced phagocytic elimination of these bacteria by alveolar macrophages in patients with chronic obstructive pulmonary disease. Furthermore, transcriptomic and proteomic analyses have shown that NRF2 directly upregulates the transcription of the scavenger receptor MARCO (macrophage receptor with collagenous structure), which increases macrophage capacity for phagocytosis (32). NRF activation in peripheral organs such as the liver and lungs is critical for protection against infections targeting these organs. For example, NRF2 activation in liver-protected mice against infection with *Lactobacillus rhamnosus* by enhancing the liver’s antioxidant pathways. Similarly, NRF2 activation promoted the expression of protective responses in alveolar macrophages during infection with *Mycobacterium tuberculosis* (33). In non-infection models, NRF2 signaling was also effective in protecting mice against liver damage caused by an overdose of acetaminophen and acute alcohol consumption. Acute liver failure caused by acetaminophen overdose, treated with compounds that induce NRF2 activation, such as taraxasterol, induces increased expression of HO-1 and antioxidant redox. Inversely, genetic ablation of NRF2 or chemical inhibition of NRF2 attenuates the anti-inflammatory effect of tarxasterol in the acetaminophen model system (34).

We recently examined the role of NRF signaling during *Ehrlichia* infection. Our data demonstrated an immune evasion mechanism where fatal disease associated with severe tissue injury following infection with highly virulent *E. japonica* is due to, in part, inhibition of NRF2 activation. Acute liver injury in *E. japonica*-infected mice was associated with reduced activation (nuclear translocation) of NRF2 signaling in hepatocytes compared to uninfected mice. Attenuated NRF2 signaling during severe ehrlichiosis in mice resulted in reduced expression of genes such as NQO1 and GPX4 that play a key role in redox balance, lipid peroxidation, and prevention of oxidative stress (3).

**Fig 1:**
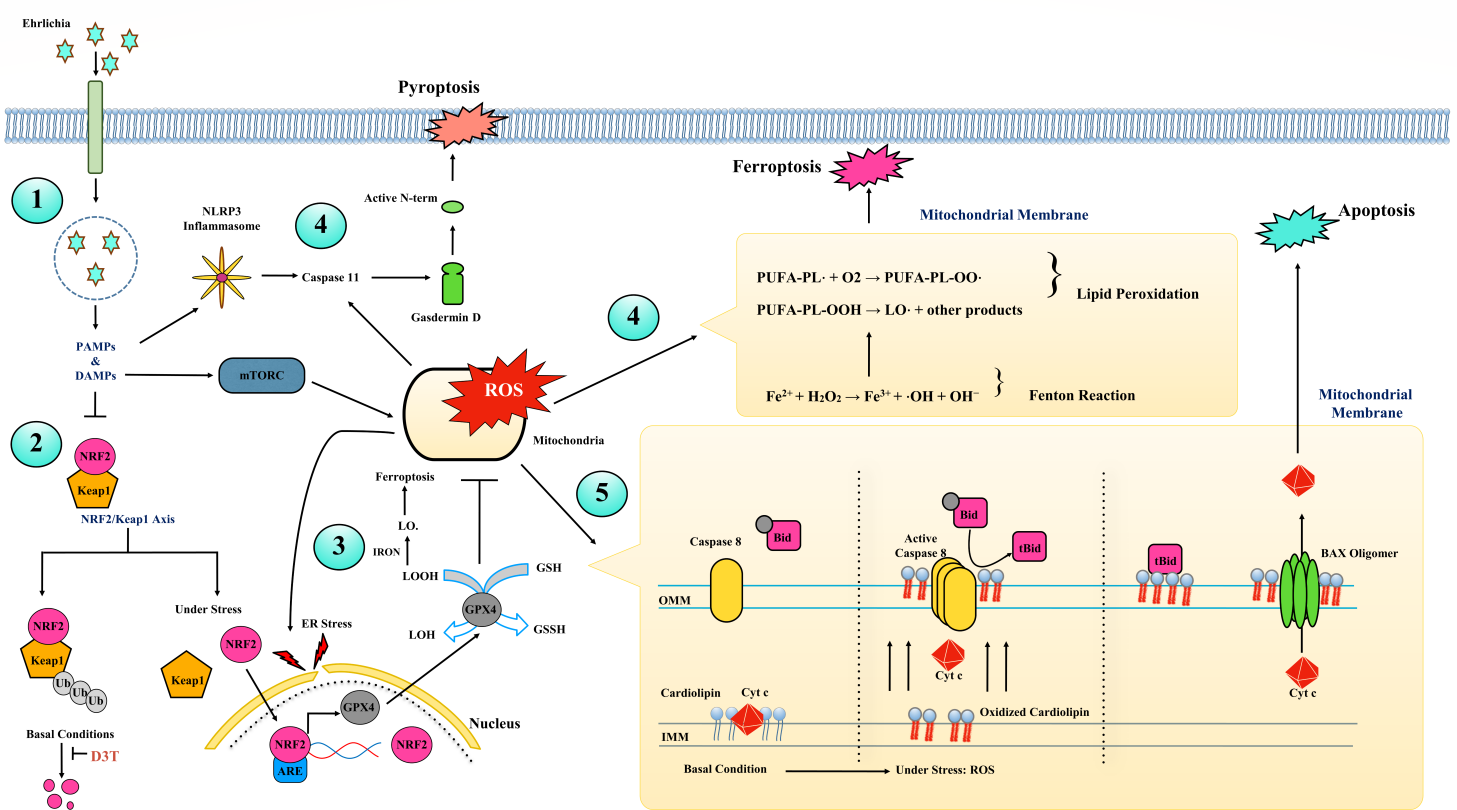
Graphical model illustrating different steps involved in oxidative damage in hepatocytes and hepatocellular death during severe ehrlichiosis. (1) *E. japonica* invasion and early host response: *E. japonica* invades target cells such as hepatocytes and resides within endosomes. (2) Inhibition of NRF2 signaling: Infection with *E. japonica* results in dysregulation of the NRF2/KEAP1 axis and inhibition of NRF2 activation, a process likely mediated by secreted PAMPs and/or DAMPs. Under basal conditions, KEAP1 interacts with NRF2 and targets it for ubiquitination, which ultimately leads to its degradation by the ubiquitin-proteasome system, 26S proteasome. However, under oxidative stress, KEAP1 gets modified and cannot target NRF2 for degradation, which causes NRF2 translocation to the nucleus and activation of NRF2 signaling. However, infection with *E. japonica* inhibits the nuclear translocation of NRF2, which leads to downregulation of downstream genes involved in antioxidant defense, detoxification, and cell survival, such as GPX4. D3T (an antioxidant thiazole derivative drug) has therapeutic effects by enhancing NRF2 expression, stabilizing cytosolic NRF2, causing its nuclear translocation, and activating anti-oxidative response. (3) Inhibition of GPX4-mediated antioxidant defense mechanism: Inhibition of NRF2 activation and decreased GPX4 expression enhance cell death by promoting ferroptosis, an iron-induced lipid peroxidation-dependent cell death. (4) Iron plays a critical role in ferroptosis by catalyzing the Fenton reaction, generating ROS. Elevated levels of ROS lead to the accumulation of misfolded proteins, then ER stress, and non-canonical inflammasome activation. ROS also initiates lipid peroxidation, resulting in the formation of lipid hydroperoxides (LOOH) and lipid alkoxyl radicals (LO•). The accumulation of LOOH and LO• causes membrane damage and finally leads to ferroptosis, an iron-induced lipid peroxidation-dependent cell death. Cytoplasmic GPX4 functions as a key antioxidant defense as it detoxifies lipid peroxides with the help of glutathione, which prevents lipid peroxidation and ferroptosis. (5) Mitochondrial damage and cardiolipin-mediated inflammasome activation: *E. japonica* induces mitochondrial damage via ROS generation in hepatocytes, leading to oxidation of cardiolipin. Oxidized cardiolipin translocates to the outer mitochondrial membrane, detaching Cyt c, recruiting pro-caspase 8, and promoting its activation. Active caspase 8 cleaves BID, a pro-apoptotic protein, to its active form tBID, activating BAX and forming a BAX oligomer that creates pores in the inner mitochondrial membrane. This promotes apoptosis by releasing cytochrome c and other pro-apoptotic factors into the cytoplasm.

Small molecules can be used to target oxidative stress in various diseases (Table 1). The key therapeutic targets to prevent oxidative stress involve the prevention of oxidant generation, blocking oxidative stress-induced signaling pathways, and inducing antioxidant defenses. Studies have shown that anti-oxidative molecules such as GPX and SOD play critical roles in host defense against oxidative stress. GPX and SOD directly neutralize deleterious ROS. Thus, targeting these critical proteins is considered a promising therapeutic strategy for diseases linked to oxidative stress. For example, ebselen, a glutathione peroxidase (GPX) mimetic, is used for Meniere’s disease in Phase II clinical trials (NCT02603081) (NCT03013400) (35). Similarly, GC4419, a superoxide dismutase (SOD) mimetic, is being evaluated in Phase I trials for squamous cell cancer (NCT01921426) (36). Also, CPUY192018, an NRF2 activator, is used to elevate the NRF2 expression and translocation, which provides cryoprotective effects by enhancing the NRF2-dependent oxidant system and reducing ROS-mediated inflammatory response (37). We have examined the effect of the restoration of NRF2 signaling during *Ehrlichia* infection using one of these compounds. Interestingly, restoration of NRF2 activation using NRF2 inducer, 3H-1,2-dithiole-3-thione (D3T), in hepatocytes infected with *E. japonica* enhanced anti-oxidative response. D3T treatment restored mitochondrial potential and decreased the total ROS produced in *E. japonica*-infected hepatocytes, which led to increased cell viability (3). In addition, treatment of *E. japonica-*infected mice with D3T prolonged mouse survival, attenuated inflammation, and enhanced bacterial clearance compared to untreated infected mice. While infected mice develop several foci of necrotic and apoptotic cells, D3T-treated and infected mice had fewer fatty changes, less necrosis, and apoptosis of hepatocytes as well as cells lining the liver sinusoid and blood vessels, including endothelial cells and macrophages. Furthermore, treatment of *E. japonica-*infected mice with D3T abrogated activation of the non-canonical inflammasome pathway marked by caspase 11 activation, which is known to play a deleterious role in severe ehrlichiosis (3). Other activators and mimics that can be used in the treatment of oxidative stress in future studies are described in Table 1 and Fig. 1.

**TABLE 1:** Comprehensive overview of antioxidant compounds, their protein targets, mechanisms of action, therapeutic applications, and clinical trial identifiers

Compound	Protein (antioxidant)	Mechanism of action	Proposed actions	Clinical trial IDs
Sulforaphane	NRF2	Activator of NRF2	FXTAS (rare genetic progressive neurodegenerative disorder), chronic kidney disease, schizophrenia, chemoprevention from cancer patients, autism spectrum disorder (38–41).	NCT05233579 NCT05153174 NCT04521868 NCT03232138 NCT02561481
Resveratrol	NRF2	Activator of NRF2	Chronic renal insufficiency, type 2 diabetes mellitus, obesity, colonic mucosa cancer, polycystic ovary syndrome (42–45).	NCT02433925 NCT01038089 NCT01412645 NCT00256334 NCT01720459
Quercetin	NRF2, free radical	Activator of NRF2, free radical scavenger	COVID, coronary artery disease progression, autism spectrum disorders (46–48).	NCT04468139 NCT03943459
Dimethyl fumarate	NRF2	NRF2 activator	Multiple sclerosis (49, 50).	NCT02683863
Oltipraz	NRF2	NRF2 activator	Non-alcoholic fatty liver disease, lung cancer prevention (51, 52).	NCT01373554, NCT00006457
N-acetyl cysteine (NAC)	GSH	GSH mimic	Neuropathy, cystic fibrosis (53, 54).	NCT01614431 NCT02252341 NCT00676195
ALT-2074	GPX	GPX mimic	Inflammatory response, neuronal death, coronary artery disease (55–57).	NCT00491543
Ebselen	GPX	GPX mimic	Meniere disease, diabetes mellitus (35, 58).	NCT02603081 NCT04677972 NCT00762671
Ethaselen	GPX	GPX mimic	Reverse cisplatin resistance, non-small cell lung cancers (59).	NCT02166242
GC4419	SOD	SOD mimic	Squamous cell cancer (36).	NCT01921426

## NRF2 AND THE TYPE I INTERFERON (IFN-I) RESPONSE IN EHRLICHIOSIS

IFN-I is a crucial component of the innate immune response during viral and bacterial infection. Elevated levels of IFN-I cytokines (IFNα and IFNβ) are produced during severe murine infection with *E. japonica*. Macrophages and plasmacytoid dendritic cells are major cellular sources of these cytokines during *E. japonica* infection. Binding of IFN-1 cytokines (IFNα and IFNβ) to IFN-I receptor on hepatocytes initiates activation of caspase 11, a non-canonical inflammasome pathway, which leads to release of high mobility group box 1 (HMGB1, a major DAMP) and inflammatory cell death known as pyroptosis. We have shown that IFNAR-deficient mice are resistant to fatal *Ehrlichia* infection compared to wild-type mice. This resistance to *Ehrlichia* infection was associated with the decreased bacterial burden and attenuated liver pathology, which was attributed to enhanced protective immunity and reduced activation of caspase 11, respectively (1, 60). How virulent *Ehrlichia* regulates the production of IFN-I cytokines remains unknown. However, mitochondrial DAMPs could be mediators of IFN-I response during severe *Ehrlichia* infection (3, 18, 61). In addition, a recent study has shown that NRF2 suppresses IFN-I response during herpes simplex virus infection (62). Thus, it is possible that virulent *E. japonica* triggers the IFN-I response via inhibition of NRF2 activation. This ultimately promotes caspase 11 activation and subsequent inflammation and cell death, causing hepatic pathology and heightened mortality in infected mice. This conclusion is also supported by the finding that the outcome of infection in *E. japonica*-infected IFNAR knockout mice that are resistant to severe ehrlichiosis mimics the host response of *E. japonica*-infected mice treated with NRF2 inducer, D3T. In both mice groups, resistance to fatal ehrlichiosis correlated with attenuation of caspase 11 activation, minimal liver pathology, lower bacterial burden (due to limited cell death and bacterial dissemination), as well as prolonged survival.

How NRF2 inhibits the IFN-I response is not completely understood. However, studies suggested that this mechanism occurs via regulating STING signaling, which is the main cytoplasmic sensor that senses viral or host DNA, triggering IFN-I signaling. Studies using NRF2-deficient human epithelial cells have demonstrated that NRF2 acts as a negative regulator of STING-dependent type I interferon responses (63). In an autoimmune lupus model, a higher activation of NRF2 causes a reduction of the IFNAR levels and alters the macrophage repolarization (64). These studies are consistent with our earlier studies showing that IFN-I responses during infection with virulent *E. japonica* are associated with M1 macrophage polarization (65). However, the influence of the NRF2-IFN-I axis on macrophage polarization and its subsequent impact on the outcome of *E. japonica* infection remains to be determined.

## GAMMA-INTERFERON-INDUCIBLE LYSOSOMAL THIOL REDUCTASE (GILT) AND POSSIBLE ROLE IN EHRLICHIOSIS

In addition to NRF2, there are other host protective proteins, including GILT, which play an essential role in cellular redox status and host responses to infections (66–68). GILT, which is also called an interferon gamma (IFNγ)‐inducible protein 30 (IFI30), is an enzyme primarily localized to the lysosome and catalyzes the reduction of disulfide bonds. GILT maintains the cellular redox state, which impacts autophagy and cellular proliferation. The absence of GILT in non-immune cells, such as fibroblasts, and immune cells, such as T cells, decreases the expression and activity of superoxide dismutase 2 (SOD2), the mitochondrial enzyme that converts superoxide to hydrogen peroxide. Deficiency of GILT in host cells increased oxidative stress by producing high levels of superoxide (66). Other studies showed that cellular GILT deficiency decreases the level of reduced glutathione while increasing the level of the oxidized form of glutathione, resulting in oxidative stress. Furthermore, GILT is critical for maintaining mitochondrial membrane potential and autophagy. Lack of GILT decreases mitochondrial membrane potential and autophagy (66). Notably, several studies have shown that *Ehrlichia* exploits beclin, a key component of autophagy initiation pathways in macrophages to obtain nutrients that promote their intracellular survival and replication. On the other hand, as a host defense mechanism, infection of macrophages with *E. japonica* triggers MYD88 signaling that inhibits autophagy induction and flux via mTORC1 activation (18). As mentioned above, the inhibition of autophagy flux (autophagosome-lysosomal fusion) leads to defective mitochondrial autophagy (mitophagy) and contributes to the accumulation of ROS, oxidative stress, and apoptosis. Although the role of GILT in this process is not yet examined, it is possible that *Ehrlichia* triggers inhibition of autophagy and mitophagy via inhibition of GILT, a mechanism that warrants further investigation.

In addition to its role in maintaining a cellular redox state, GILT plays a pivotal role in the induction and activation of adaptive CD4 and CD8 T-cell responses via promoting MHC class I and class II antigen presentation, respectively. GILT is constitutively expressed by antigen-presenting cells, such as B cells, dendritic cells, and macrophages, and IFNγ upregulates its expression in these cells. During viral infection with herpes simplex virus, GILT enhances the cross-presentation of viral antigen to CD8 T cells, leading to cross-priming and activation of cytotoxic CD8 T cells (66). We have shown that severe and potentially fatal ehrlichiosis is mediated, in part, by pathogenic cytotoxic CD8 T cells. As *Ehrlichia* resides in the phagosomal compartment and does not access the cytosol and the MHC class I classical pathway of antigen presentation, we believe that induction of CD8 T cells during *Ehrlichia* infection is via cross-presentation by dendritic cells. Whether GILT/IFI30 is involved in the induction of this pathogenic adaptive immune response during severe and fatal ehrlichiosis remains elusive.

## ER STRESS AND ITS LINK TO OXIDATIVE STRESS DURING SEVERE EHRLICHIOSIS

Endoplasmic reticulum (ER) is a key cell organelle involved in multiple cellular functions, including protein synthesis, intracellular transport of lipids and proteins, and storage and release of calcium. Protein degradation in the ER is a critical event that maintains cellular homeostasis. Under ER stress conditions, misfolded proteins cannot be adequately removed by the cell’s protein-folding machinery and thus accumulate in the cells, which results in an adaptive response referred to as unfolded protein response (UPR). To restore cellular homeostasis, this UPR inhibits protein synthesis and regulates several genes involved in cellular transcription and cell survival. The UPR is initiated and regulated by three ER sensors, including inositol-requiring enzyme 1 (IRE1), protein kinase R-like ER kinase (PERK), and activating transcription factor 6 (ATF6) (69). Under normal conditions, these sensors remain inactive as they are bound to BiP (an Hsp70 family member). However, when there is an increased presence of misfolded proteins, BiP dissociates from these sensor proteins, triggering UPR (69). The genes of UPR are linked to oxidative stress, autophagy, and mitochondrial dysfunction (70). Increased evidence indicates that oxidative stress and ER stress are crucial in the pathology of several infectious and non-infectious diseases such as Alzheimer’s disease, atherosclerosis, chronic kidney disease, neurodegenerative disorders, and cancers (71).

Our recent studies showed that virulent *E. japonica,* which causes acute liver injury and fatal disease in mice, triggers oxidative stress and ER stress in hepatocytes (3, 18). Infection of mice with *E. japonica* induced elevated mRNA expression of several ER sensors, including PERK, IRE1, ATF6, XBP1, XBP1s, and CHOP, in the livers of infected mice compared to controls (3). Fatal *E. japonica* infection also triggered increased expression of ER-associated degradation (ERAD) genes, including EDEM, GADD34, and DR5, in livers of infected mice when compared to livers from uninfected or non-fatal infection with a mildly virulent *E. muris* strain. *In vitro,* studies demonstrated oxidative/ER stress response in primary murine hepatocytes following infection with *E. japonica* that mimics the *in vivo* response in liver tissues of *E. japonica-*infected mice. This UPR was associated with significant distortion and disorganization of ER and mitochondria in *E. japonica*-infected liver tissues. Interestingly, although mild infection with *E. muris* did not trigger significant expression of genes involved in UPR compared to uninfected mice, the liver tissues from these mice exhibited distortion of ER, although mitochondrial cristae and structure were preserved (3). Mechanistically, we found that *E. japonica*-induced inhibition of NRF2 signaling (antioxidant response mechanism) contributes to ER stress in infected hepatocytes (3). Inversely, ER stress following severe *E. japonica* infection mediates the suppression of NRF2 expression/activation in infected hepatocytes as a positive feedback loop (3). Consistent with other infection models, such as that caused by *Mycobacterium tuberculosis*, ER stress in hepatocytes during severe ehrlichiosis promotes host cell death via apoptosis (72). Although the exact mechanism that triggers apoptosis following ER stress in *E. japonica*-infected cells is not yet known, other studies have shown that ER stress during infection with *M. tuberculosis* causes apoptosis via toll-like receptor 2/4 signaling following binding to a 38 kDa antigen (72). Other mycobacterial factors also modulate the host signaling, assisting in colonizing the host (73–75). Nevertheless, our studies, as well as other studies using different infection models, illustrate the complex interplay between ER stress and oxidative stress that involves various microbial metabolites triggering inflammation and tissue injury (72, 76, 77). A comprehensive understanding of ER and antioxidant signaling pathways during *Ehrlichia* infection is crucial for developing effective therapeutic strategies.

## CONCLUSION AND FUTURE DIRECTION

**Fig 2:**
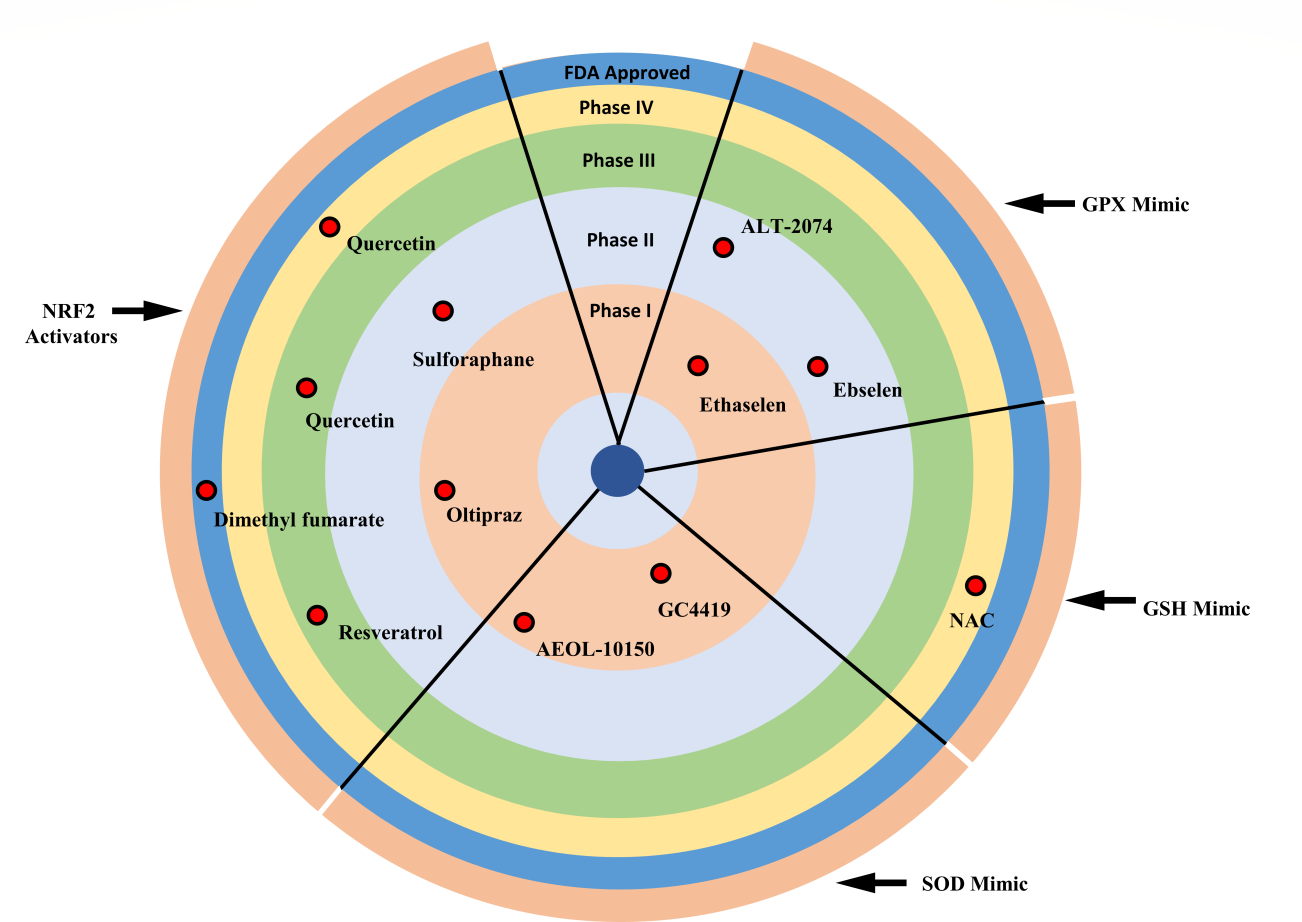
Bull’s eye representation of antioxidant drug candidates in different phases of clinical trials: Phase 1 (orange), Phase 2 (light blue), Phase 3 (green), and Phase 4 (yellow), and FDA-approved drugs (blue). Drug compounds are additionally classified based on their specific target proteins such as NRF2 activators, SOD mimics, GPX mimics, and GSH mimics. For additional details, including antioxidant compounds, their protein targets, mechanisms of action, therapeutic applications, and clinical trial identifiers, refer to Table 1.

Oxidative stress is a pivotal factor in the pathogenesis of *Ehrlichia*-induced sepsis. *Ehrlichia* triggers several mechanisms that account for the development of oxidative stress, as summarized in Fig. 1. Targeting key molecules or pathways that account for oxidative stress and tissue damage is a promising therapeutic approach. Various antioxidant drugs are in the initial stages of development (Phase 1 and Phase 2) (Fig. 2). Some of these drugs, such as dimethyl fumarate, an approved drug for psoriasis and multiple sclerosis, can be repurposed and used as therapeutic drugs that inhibit oxidative stress in infectious diseases such as ehrlichiosis, where multi-organ dysfunction and potentially fatal sepsis is due to defective anti-oxidative response (Table 2). Furthermore, potential approaches include developing selective antioxidants that target mitochondrial or cytoplasmic oxidants, using nanoparticles to deliver antioxidants directly to infected cells that enhance drug efficacy, and employing combination therapies that provide synergistic effects of the treatment. Implementation of these new therapeutic strategies may also contribute to a deeper understanding of the role of oxidative stress, not only during severe ehrlichiosis but also for other infectious and non-infectious diseases.

**TABLE 2:** Different analogs of dimethyl fumarate are available that can be further used in clinical settings^*a*^

Name	Chemical structure	Functions
Dimethyl itaconate	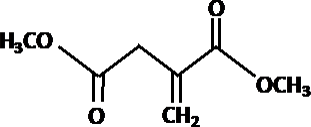	Induces trained immunity in human monocytes to protect mice against infection. Protects against anti-mycobacterial response (78, 79).
Ethyl pyruvate	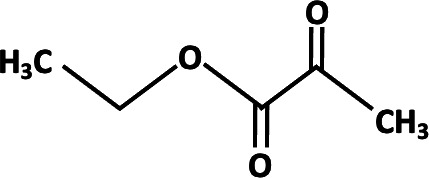	Played a protective role in spinal cord injury. Protection of multiple organ ischemia-reperfusion injury (80).
Diroximel fumarate	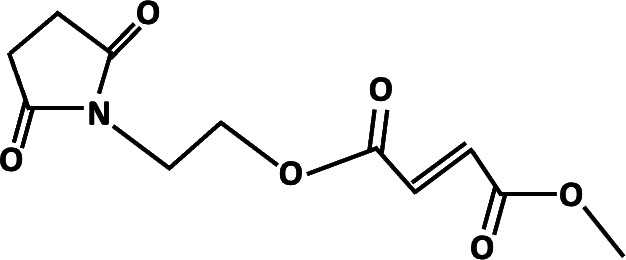	Acts as immunomodulators that are primarily used to treat multiple sclerosis (81).
Monomethyl fumarate	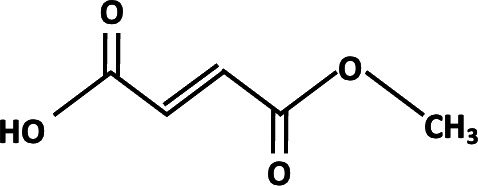	Treatment of relapsing forms of multiple sclerosis (82).
Tetraethylthiuram disulfide	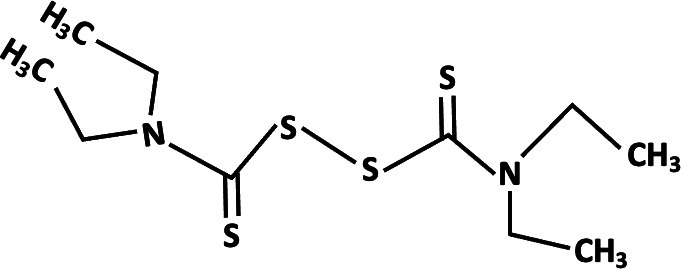	Treatment of chronic alcoholism and inhibition of *Plasmodium falciparum* (54, 83).

^*a*^ Dimethyl fumarate is an FDA-approved drug for the treatment of psoriasis and multiple sclerosis.
